# Characterizing changes to individual-specific brain signature with age

**DOI:** 10.3389/fnagi.2025.1493855

**Published:** 2025-07-02

**Authors:** Monireh Taimouri, Vikram Ravindra

**Affiliations:** Department of Computer Science, University of Cincinnati, Cincinnati, OH, United States

**Keywords:** brain signature, functional connectome, matrix sampling, biomarkers of aging, feature selection

## Abstract

The increasing prevalence of neurodegenerative diseases in an aging population underscores the critical need for reliable biomarkers distinguishing normal aging from pathological neurodegeneration. This study leverages neuroimaging to identify age-resilient biomarkers, establishing a baseline of neural features that are relatively stable across the aging process. Our research objectives are threefold: (a) Validate a methodology using leverage scores to identify age-robust neural signatures; (b) Confirm the consistency of these features across a diverse age cohort (18-87 years); and (c) Assess the stability of individual-specific neural characteristics across multiple brain parcellations (Craddock, AAL, and HOA). Using functional connectomes data from resting-state and task-based fMRI, we found that a small subset of features consistently capture individual-specific patterns, with significant overlap (~50%) between consecutive age groups and across atlases. Our approach effectively minimized inter-subject similarity while maintaining intra-subject consistency across different cognitive tasks. The stability of these signatures throughout adulthood and their consistency across different anatomical parcellations provide new perspectives on brain aging. They highlight both the preservation of individual brain architecture and subtle age-related reorganization. These findings enhance our understanding of age-related brain changes, potentially aiding in differentiating normal cognitive decline from neurodegenerative processes.

## 1 Introduction

Research on aging and its impact on the brain is a rapidly growing field, driven by the increasing prevalence of age-related neurodegenerative diseases and the increasing global population of older adults. Identifying biomarkers that accurately delineate the biological processes of aging is a cornerstone in the quest to understand and mitigate age-related decline. Such biomarkers hold the potential to predict functional outcomes and distinguish between the effects of normal aging and pathological neurodegeneration. Baker and Sprott (1988) set forth criteria for ideal biomarkers, which have been expanded to emphasize the need for reproducibility, minimal invasiveness, and, in particular, resistance to confounding age-related factors, as supported by recent discourse (Fuellen et al., 2019).

The inherent heterogeneity and complexity of aging present significant challenges in identifying a universal biomarker. In light of this, the field is pivoting toward a multi-biomarker approach that can provide a richer, more integrated characterization of aging, as proposed by Earls et al. (2019) and Kudryashova et al. (2020). The utility of composite biomarkers, as demonstrated by Belsky et al. (2018) and Hastings et al. (2019), further underscores the potential of nuanced indicators to enhance predictive power for outcomes related to aging.

A pivotal study by Jiang et al. (2022) has refined our understanding of brain network organization, revealing that distinct but overlapping functional connectivity patterns can predict cognitive function and age-related changes. They demonstrate that while cognitive decline and aging affect all network connections, specific networks, such as the dorsal attention network, show a unique relationship with cognitive performance independent of age. This insight is crucial for differentiating between cognitive decline due to normal aging and neurodegenerative processes.

Building on the current methodologies in aging and neuroimaging research (Jiang et al., 2022; Setton et al., 2023; Zheng et al., 2023), this study uses functional neuroimages to uncover biomarkers that are resilient to age-related changes. Identifying consistent signals over the lifespan allows us to establish a baseline of neural features that are relatively unaffected by aging. This baseline is crucial to discern neural alterations attributable to neurodegenerative diseases, distinct from normal aging effects.

Our objectives are threefold: (a) To validate a methodology using leverage scores to identify robust neural signatures against age-related variability. (b) To determine the consistency of these neural features within a diverse age cohort and to evaluate their resilience to aging-related changes. (c) To assess the stability of individual-specific neural characteristics across multiple parcellations to fortify our findings.

## 2 Materials and methods

We initiate this section with a brief description of relevant software infrastructure—the CamCan dataset and pertinent brain atlases. We then provide details for the setup and methodology we used in this study. Finally, we discuss the significance and open questions posed in related research articles.

### 2.1 Dataset and atlases

In this paper, we use the dataset collected by the Cambridge Center for Aging and Neuroscience (Cam-CAN). Specifically, we show results from the Cam-CAN Stage 2 study cohort, a substantial resource for investigating age-related changes in cognition and brain function. Cam-CAN Stage 2 includes a diverse range of data modalities, including Magnetic Resonance Imaging (MRI)—both structural and functional, Magnetic Encephalography (MEG), and cognitive-behavioral data collected from a cohort of 652 individuals (322 males and 330 Females) spanning the adult lifespan (from 18 to 88 years). As this dataset samples from a substantially diverse population, it allows for a comprehensive characterization of healthy cognitive aging processes, specifically focusing on understanding age-related alterations in brain structure, function, and cognitive performance.

For our analysis, we incorporate three distinct brain atlases, consisting of two anatomical atlases and one functional parcellation. The atlases are as follows: (a) the *Automated Anatomical Labeling (AAL)* atlas (Tzourio-Mazoyer et al., 2002) with 116 regions, (b) the *Harvard Oxford (HOA)* atlas (Makris et al., 2006) with 115 regions, and (c) the *Craddock* atlas (Craddock et al., 2012) which provides a more granular functional parcellation with 840 regions. Functional parcellation involves segmenting the human brain into distinct territories or regions based on the functional aspects of neural activity. Unlike anatomical divisions, based on physical brain structures, functional parcellation focuses on how different brain regions are involved in specific processes and functions. It is worth noting that the choice of functional parcellation can have a substantial impact on how researchers approach the study and modeling of brain functions, as well as how they analyze and interpret data related to neural activity and mental processes (Varoquaux et al., 2019).

### 2.2 Methodology

#### 2.2.1 Pre-processing

The functional MRI data utilized in this study underwent artifact and noise removal by the dataset curators. Separate pipelines were tailored for processing resting-state, movie-watching, and sensorimotor (SMT) tasks. We included all participants with available resting-state and task-based fMRI scans who passed the original Cam-CAN quality control. The Cam-CAN project, as reported in Shafto et al. (2014) and Taylor et al. (2017), did not apply a framewise displacement threshold to exclude participants based on head motion during fMRI scans. Instead, a rigorous pre-screening process ensured that participants could comfortably lie still during scanning, and foam cushions were used during acquisition to minimize movement. Quality control procedures were applied *post-hoc* to all imaging data to ensure integrity, but no additional participant-level exclusions were performed in our study based on motion metrics. As part of the standard Cam-CAN preprocessing pipeline, functional MRI data were processed using the SPM12 software and the Automatic Analysis (AA) framework. The following motion correction steps were applied: (a) Realignment (rigid-body motion correction) to correct head motion within sessions, (b) Co-registration of functional scans to each subject's T1-weighted anatomical image, (c) Spatial normalization to MNI space using Diffeomorphic Anatomical Registration Through Exponentiated Lie Algebra (DARTEL) templates, and (d) Smoothing with a 4mm FWHM Gaussian kernel.

The output of the aforementioned preprocessing pipeline is a clean, functional MRI time-series matrix **T** ∈ ℝ^*v* × *t*^, where *v* and *t* denote the number of voxels and time points respectively. Next, we parcellate each **T** to create a region-wise time-series matrix **R** ∈ ℝ^*r* × *t*^ for each of the three atlases mentioned previously. Here, *r* represents the number of regions. Then, we compute the Pearson Correlation matrices (**PC**) for each of these region-wise time-series matrices, where **C** ∈ [−1, 1]^*r* × *r*^. Each (*i, j*)-th entry represents the strength and direction of the correlation between the *i*-th and *j*-th regions. In the literature, these undirected correlation matrices are also called Functional Connectomes (FCs).

To prepare the data for group-level analysis, we vectorize each subject's FC matrix by extracting its upper triangle (since correlation matrices are symmetric with ones on the diagonal, we use only the upper triangular part) and stack these vectors to form population-level matrices for each task (e.g., **M**_rest_, **M**_smt_, and **M**_movie_). Each row in these matrices corresponds to an FC feature, and each column corresponds to a subject. For age-specific analysis, we partition the subjects into non-overlapping age cohorts and extract the corresponding columns to form cohort-specific matrices of shape [*m* × *n*], where *m* is the number of FC features and *n* is the number of subjects in the cohort. Leverage scores are then computed for each cohort matrix to identify high-influence FC features that capture population-level variability within each age group.

#### 2.2.2 Feature selection and leverage score sampling

The goal of this paper is to find a small set of regions that strongly code for individual-specific signatures that remain stable across ages. However, the number of rows in the **C** matrices computed previously is $O\left(r^{2}\right)$, which can become prohibitively large for fine-grained parcellations. Thus, our next step is to identify a subset of features, those with non-zero values in the correlation matrix, which provide the most insight into individual signatures. These selected features have clear physical interpretations, representing the edges of functional connectomes. By isolating a small, yet informative subset, we can better capture individual differences while maintaining interpretability. Since each of these selected features is associated with two regions of the brain (corresponding to the two nodes of each edge), we can directly map them to the spatial domain, facilitating further analysis of their anatomical significance.

As interpretability is a core requirement, our method is based on leverage-score sampling. Consider again **M** as the data matrix representing connectomes. Let **U** denote an orthonormal matrix spanning the columns of **M**. The leverage scores for the *i*-th row of **M** are defined as the two-norm of the same row in **U**. i.e.,


(1) $$\begin{array}{l} l_{i}={\text{U}}_{i,⋆}{\text{U}}_{i,⋆}^{T},\;\forall i\in \left\{1,\ldots ,m\right\}. \end{array}$$

The values of the leverage scores themselves are a measure of the relative importance of different rows. Rows with higher scores have more “leverage” than rows with lower scores. While traditional approaches use leverage scores in a randomized manner, we simply sort the scores in descending order and retain only the top *k* features. The theoretical guarantees for our deterministic strategy are provided by Cohen et al. (2015). We note that we have previously used leverage-score sampling in Ravindra et al. (2021) to find individual-specific signatures in healthy young adults from the Human Connectome Project (HCP) dataset, where pairs of images corresponding to the same individual were matched with over 90% accuracy. A key advantage of the method was that it required the acquisition of only one image for every subject, whereas other methods such as Finn et al. (2015), Byrge and Kennedy (2019) and Gratton et al. (2018) required at least two sessions.

### 2.3 Related literature

#### 2.3.1 Individual specific signature

Neuroimaging research has increasingly focused on the individuality of brain connectivity, with resting-state fMRI providing a window into the unique neural wiring of each person. The foundational work by Mueller et al. (2013) highlighted the diversity of functional connectivity patterns across individuals, with particular variability in regions associated with higher cognitive functions and evolutionary development. Building on this, Finn et al. (2015) deconstructed the identification task at both the whole-brain and the network-specific levels, achieving an impressive 93% success rate through the utilization of region-wise correlation matrices encompassing the entire brain. Notably, certain networks, such as the medial frontal and frontoparietal networks, emerged as highly discriminative, echoing findings of Mueller et al. (2013) and Miranda-Dominguez et al. (2014), although with a prerequisite for prior knowledge of functional brain regions. In a comparative dialogue, Airan et al. (2016) and Byrge and Kennedy (2019) also explored the identifying power of certain brain connections. Airan et al. (2016) optimized neuroimaging techniques to differentiate individual subjects, finding key acquisition times and brain regions that maximize between-subject variability and minimize within-subject variability, enhancing the design and interpretation of resting-state fMRI experiments. Byrge and Kennedy (2019) advanced this domain by introducing a technique known as connectome fingerprinting, which could reliably identify individuals based on a select few functional connections, significantly reducing the complexity from tens of thousands to merely dozens while maintaining identification accuracy. In 2017, using Human Connectome Project data, Finn et al. (2017) examined how different brain states, beyond resting conditions, may better highlight individual differences in functional connectivity. They analyze variability across conditions to improve biomarker discovery and individualized brain mapping, setting the stage for future research. After that, Gratton et al. (2018) added depth to the understanding of individual brain signatures by examining the temporal consistency of connectivity patterns, reinforcing the notion that functional networks are reflective of stable individual differences over time. Diversifying the methodological landscape, Hannum et al. (2023) utilized Linear Discriminant Analysis to achieve near-perfect accuracy in identifying individuals from a large cohort. Their work also scrutinized the influence of image preprocessing on cognitive state decoding and pinpointed specific sub-networks that are vital for accurate identification.

#### 2.3.2 Biomarkers of aging

Understanding the biological underpinnings of aging, particularly in the brain, is pivotal for addressing age-related cognitive decline. Recent neuroimaging studies have revealed consistent individual differences in brain function, with connectivity patterns in medial frontal and frontoparietal networks showing remarkable stability over time, as demonstrated by Horien et al. (2019). These findings underscore the potential for personalized approaches to track and intervene in the aging process. Building on this, Higgins-Chen et al. (2021) proposes a hierarchical framework that incorporates epigenetic and neuroimaging-derived biomarkers, offering a multifaceted view of the aging brain. These biomarkers not only reflect biological aging but also possess a significant relationship with cognitive and mental health outcomes. Hartmann et al. (2021) extends this discussion by evaluating general biomarkers of aging and cellular senescence, adopting the PICO(Population, Intervention, Comparison, Outcome) strategy to elucidate the discrepancies between chronological and biological aging. This work is instrumental in refining the biomarkers used to monitor aging's impact, with implications for both clinical practice and aging research. Zamani Esfahlani et al. (2022) contributes to this discussion by examining age-related changes in the coupling between brain structure and function, noting a general decline across the lifespan with specific reductions in sensorimotor regions. Conversely, areas tied to higher cognitive functions exhibit more resilience to aging effects, suggesting a nuanced landscape of neural aging. Jiang et al. (2022), leveraging data from the CamCAN cohort, further our understanding of cognitive decline with age, linking it to alterations in brain network connectivity. This research supports theories of neural dedifferentiation and compensatory mechanisms, which may guide future strategies to mitigate cognitive aging. Lastly, the collaborative effort by the Aging Biomarker Consortium (ABC) (Consortium et al., 2023) highlights the field's collective endeavor to standardize brain aging biomarkers. Their consensus paves the way for a systematic assessment of brain aging, with potential benefits for the development of targeted interventions and treatments for aging-related brain diseases. These studies form a mosaic of insights into the complex interplay between aging, brain structure and function, and cognitive health, pointing toward an increasingly personalized approach to managing and understanding aging.

#### 2.3.3 Feature selection methods

Feature selection is a pivotal process in machine learning and pattern recognition, with significant implications for brain imaging studies aimed at elucidating brain signatures of cognitive processes (Popp et al., 2024), disease states (Guo et al., 2017; Shi et al., 2020), and other neurological phenomena. In the domain of disease diagnosis, Guo et al. (2017) harnessed deep neural networks to parse resting-state fMRI data, unveiling 32 functional connections as potential biomarkers for Autism Spectrum Disorder (ASD). Building on this, Shi et al. (2020) improved the specificity of feature selection by creating a minimum spanning tree from connectivity data, thus refining ASD classification by balancing discriminative power and feature redundancy. Furthering our understanding of cognitive function, Xu et al. (2020) compared discrimination-based and reliability-based feature selection methods using the Human Connectome Project's extensive dataset. Their findings suggest discrimination-based features excel in decoding brain states, while reliability-based features demonstrate enhanced stability–a crucial consideration for longitudinal studies. Rastegarnia et al. (2023)'s investigation into personalized brain decoding challenges conventional group-level models, revealing the potential for individualized machine learning models to achieve comparable accuracy with significantly less data. This work opens avenues for bespoke brain state analysis that could revolutionize cognitive neuroscience. Complementing this, Popp et al. (2024) linked individual variations in cognitive ability to structural-functional brain network coupling, validated across independent samples, providing a predictive framework for cognitive performance based on connectivity patterns.

## 3 Results

### 3.1 Result 1: sampled feature-space demonstrates a high level of individual-specific similarity for all age groups

A feature representation designed to maximize individual-level differences has two properties: (a) similarity metrics (such as correlation) between pairs of distinct individuals must be low, and (b) similarity between different pairs of images corresponding to the same subject should be high. In our first set of results, we will establish that our approach of sampling features based on their leverage scores efficiently characterizes individual-specific signals for subjects in all age groups.

We represent each fMRI session of every subject as a vectorized FC obtained from the Craddock parcellation. Next, we arrange the subjects by their age into cohorts of 50. Each cohort represents approximately five to eight years of biological age. For each cohort, we create three task-specific matrices for each rest, sensorimotor (SMT), and movie viewing. Using the rest matrix, we select 1,000 features with the most significant leverage scores from a pool of 352,380 candidate features. To demonstrate that our approach selects a parsimonious subset of features to minimize inter-subject similarity, we compute the pairwise Pearson Correlation between (a) pairs of subjects performing the same task and (b) pairs of FCs corresponding to the same subject performing different tasks. Our results show that the inter-subject within-task similarity is, on average, 0.0466 for rest, 0.0440 for sensorimotor, and 0.0340 for movie viewing. On the other hand, limiting the feature space of the top leverage scores of rest, we report within-subject similarity of 0.4492 for rest and sensorimotor and 0.4030 for rest and movie. Thus, our top features increase dissimilarity across subjects performing the same task and increase similarity within each subject, even while performing different tasks.

To show the statistical significance of the feature selection mechanism, we repeat the process for (a) all available features and (b) random subsets of features. For random features, we repeated the random selection process for a million runs and computed all previously mentioned similarity measures for each run. Our results, summarized in Tables 1, 2 demonstrate that our results are indeed statistically significant with a *p*-value of < 1*e* − 8. We have provided the same results separately for each cohort in Supplementary Tables S1, S2.

**Table 1:** This table shows the average similarity between pairs of subjects when they are performing one of the three tasks (Rest, SMT, and Movie).

**Task**	**Feature set**		
	**All**	**LS**	**Random**
Rest	0.2461 ± 0.0144	0.0466 ± 0.0107	0.2457 ± 0.0190
SMT	0.2331 ± 0.0183	0.0440 ± 0.0088	0.2329 ± 0.0217
Movie	0.1450 ± 0.0222	0.0340 ± 0.0075	0.1448 ± 0.0233

In each case, we present results for (a) all features, (b) top 1,000 leverage-score (LS) features, and (c) randomly selected features. The consistent low similarity for top LS shows that our feature selection approach ensures high dissimilarity across subjects. Here, we use Pearson Correlation as a measure of similarity.

**Table 2:** This table shows the average similarity between pairs of FCs drawn from the same subject performing different tasks.

**Task-pairs**	**Feature set**		
	**All**	**LS**	**Random**
Rest-SMT	0.4576 ± 0.0202	0.4492 ± 0.0304	0.4572 ± 0.0220
Rest-Movie	0.4343 ± 0.0200	0.4030 ± 0.0204	0.4340 ± 0.0221

In each case, we present results for (a) all features, (b) top 1,000 leverage-score (LS) features, and (c) randomly selected features. When compared to Table 1, the higher PCs for LS features demonstrate that within-subject similarity is strongly encoded in our top features.

In consistence with previous studies, we repeated the exclusion procedure described by Geerligs et al. (2016), and identified regions with insufficient coverage. Following their method, we examined the spatial coverage of each ROI by generating a functional brain mask for each participant, thresholded at 70% of the mean signal intensity. ROIs that had less than 50% overlap with the functional mask in even one subject were excluded from further analysis. This process led to the exclusion of 35 ROIs, resulting in a final set of 805 regions retained for the Craddock-840 parcellation. On examining the results obtained from this reduced atlas, we can report that the results before and after the exclusion procedure are very similar. We have included a list of excluded ROIs and pairwise similarity results in Supplementary Tables S3, S4.

While these results are similar to our earlier results in Ravindra et al. (2021), we include them for the following reasons. The earlier results were demonstrated on the HCP young adult dataset. Here, we show that our procedure is generalizable to a different dataset with a distinct age range, acquisition protocol, and parcellation scheme. Further, our following result shows that these feature sets are stable across age groups. Hence, we must first establish that they are valid representations of signatures.

### 3.2 Result 2: individual-specific signature remains stable across age

In the second phase of our analysis, we evaluate the consistency of individual-specific signatures across the adult human lifespan (ages 18–87). We generate vectorized FCs for each Craddock, AAL, and HOA parcellation to ensure reproducibility.

Subjects were sorted in ascending order by age and partitioned into non-overlapping cohorts of 50 subjects. As before, each cohort represents approximately five to eight years of biological age. To maintain comparability across different parcellations/atlases, we retain roughly 1.5 percent of the total features for each parcellation with the most significant leverage scores. This resulted in 7,000 features from 352,380 candidates for Craddock, 100 features from 6,670 candidates for AAL, and 100 features from 6,555 for HOA. We then calculate the intersection of top features between all pairs of cohorts. The intersection set's size measures the features' stability as they age. Features consistently present in cohorts across age groups are individual-specific biomarkers robust to aging.

**Figure 1:**
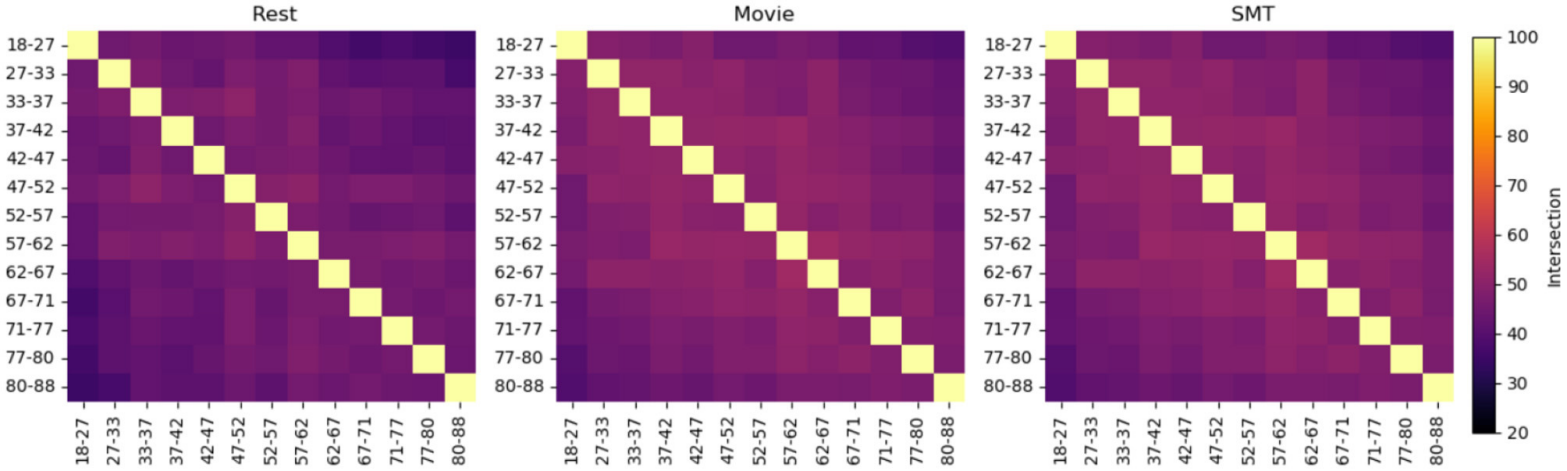
These heatmaps show the percentage intersection of top features selected using the leverage score method between age groups in the Craddock atlas, for three tasks: Rest, Movie, and SMT. Each cell represents the percentage of shared top features between two age groups, with warmer colors indicating higher overlap. The subjects are divided into non-overlapping subsets of 50 individuals, arranged by age. The diagonal represents 100% self-intersection. This visualization reveals the consistency of individual-specific neural signatures across different age ranges and cognitive states, demonstrating how brain connectivity patterns evolve or remain stable throughout adulthood under various task conditions. The average percentage intersection across age groups is 42.7% for Rest, 40.4% for Movie, and 46% for SMT.

**Figure 2:**
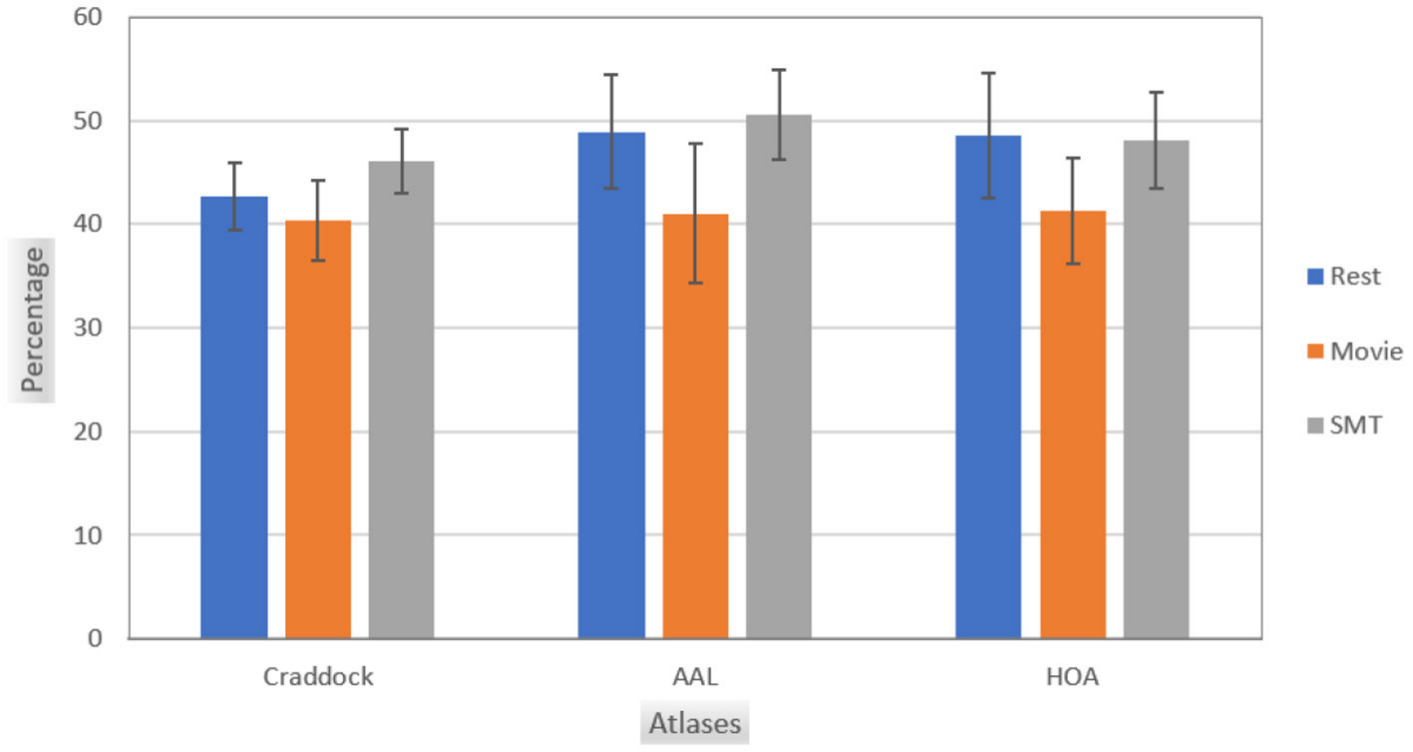
This chart shows the average intersection of feature sets between all pairs of age sub-cohorts for each brain atlas (Craddock, AAL, and HOA) and different tasks (Rest, Movie, and SMT). Each bar indicates the mean overlap of top leverage score-selected features between non-overlapping age groups, demonstrating the consistency of individual-specific neural signatures across the adult lifespan (ages 18–87). Higher values suggest greater stability of brain connectivity patterns across different age ranges.

Figure 1 illustrates the intersections of top features selected using the leverage score method between non-overlapping consecutive age groups in the Craddock atlas for three tasks: Rest, Movie, and SMT. Similar heatmaps for AAL and HOA atlases are provided in Supplementary Figure S1. Our analysis reveals consistent patterns across resting-state and task-based fMRI conditions. In resting-state fMRI, we observe consistent features with substantial overlap between consecutive sub-cohorts across all brain atlases. AAL-Rest and HOA-Rest data show approximately 50% shared features, while Craddock-Rest demonstrates an average of 43% shared features. In Task-based fMRI, Similar patterns of feature consistency are observed in movie-watching and sensorimotor task conditions. Figure 2 provides a comprehensive overview of the average feature overlap across all sub-cohort pairs for each atlas and fMRI condition. While our analysis primarily focuses on the stability of individual-specific brain signatures across ages, we also considered the influence of gender differences. Our comparison of male and female subjects across the three tasks (Rest, Movie, and SMT) reveals slight differences in feature stability, with males generally showing slightly higher stability in the SMT task than females. However, these differences are not substantial. The average percentage intersection across age groups is 30.8% for males and 30.5% for females in Rest, 30.8% for males and 31% for females in Movie, and 35.3% for males and 32% for females in SMT. Plots illustrating these gender-based comparisons in the Craddock parcellation are provided in Supplementary Figure S2.

To assess the statistical significance of these results, we selected random features as the top features for each cohort and computed the pairwise intersection as before. We repeated this experiment for one million trials. This analysis yielded an average overlap of 1.95%. Thus, the overlaps with leverage-score features are significant with a *p*-value < 1*e*−6, substantiating the robustness of our findings.

**Figure 3:**
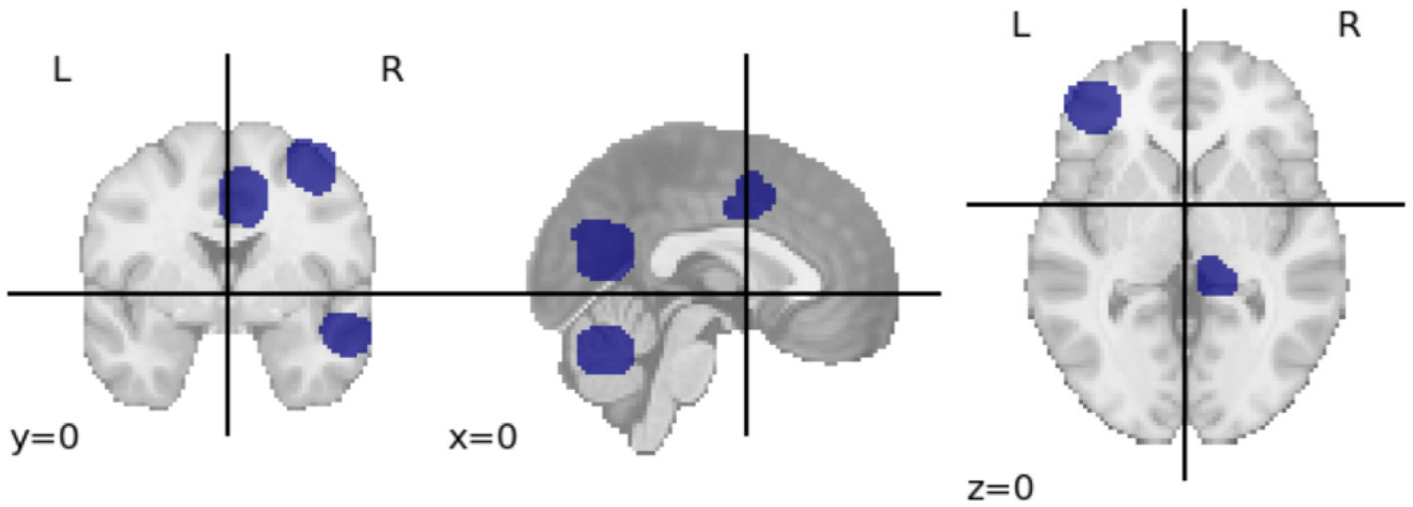
The image shows three different views (coronal, sagittal, and axial) of the brain with regions highlighted in blue. These highlighted areas represent the stable brain regions identified using the Craddock parcellation, which remained consistent across different age groups. This visualization was created using Nibabel (Brett et al., 2024).

The observed consistency in feature sets across age groups provides a foundation for investigating age-related changes. Specifically, it facilitates the identification of brain regions and their associated consistent features that are resilient to aging. To identify these regions, we first extract the common features across all cohorts using the Craddock atlas. We then calculate the brain regions corresponding to these features, extract their coordinates and match them with the AAL atlas. The resulting regions are *Middle Frontal Gyrus, Supplementary Motor Area, Middle Cingulate, Parahippocampal Gyrus, Inferior Parietal Gyrus, and Lobules 6-9 of Vermis (Cerebellum)*. Figure 3 shows the cross-sectional view of these regions in Craddock parcellation. We have also included a network visualization of these regions in Supplementary Figure S3. While these results suggest time-series signals associated with these regions are relatively resilient to aging, further study is required to understand *why* this is indeed the case.

To further validate the age-invariance of the identified stable features, we performed a statistical analysis of their relationship with age. We first extracted the shared features across all age cohorts and conducted a linear regression analysis. The results revealed that 425 out of 450 shared features (94.4%) showed no significant association with age after FDR correction (α = 0.001), confirming their statistical robustness against aging effects. Additionally, we evaluated whether these stable features could predict chronological age using a 10-fold cross-validation framework with a linear regression model. The model trained on the stable features yielded a mean absolute error (MAE) of 20.22 ± 2.18 years, indicating poor predictive performance. In contrast, models trained on 1,000 randomly selected feature sets achieved significantly better age prediction performance, with an average MAE of 12.72 ± 0.59 years. These results confirm that the stable features capture individual-specific patterns that are largely age-resilient, reinforcing their role as biomarkers of intrinsic brain organization rather than age-driven change.

Our findings align with research showing that age-related brain changes vary across regions, with some areas exhibiting relative stability. Frontal and parietal regions show gradual decline, while medial temporal structures age more rapidly (Sele et al., 2020). Despite age-related volume loss in the middle frontal gyrus (MFG), evidence suggests that compensatory mechanisms help preserve its functional role (Rajah et al., 2011). Additionally, while posterior medial temporal regions, such as the parahippocampal gyrus, are more vulnerable to aging, anterior regions remain more stable (Hrybouski et al., 2023). These findings support the idea that some regions retain individual-specific connectivity patterns, likely due to intrinsic stability and neural adaptation over time.

These results demonstrate that, while individual-specific brain signatures remain largely stable across the lifespan, age-related factors also exist. This balance between consistency and change provides valuable insight into the neurobiological underpinnings of cognitive aging, potentially revealing both preserved and altered brain networks throughout life.

### 3.3 Result 3: cross-atlas consistency of selected functional connectivity features

In the final phase of our analysis, we investigate the consistency of individual-specific neuroanatomical signatures across the Craddock, AAL, and HOA atlases. This examination is crucial for validating the robustness of our findings and assessing the generalizability of individual brain signatures across different anatomical parcellations.

We employ the following methodology to assess the consistency of individual-specific neuroanatomical signatures across atlases. First, we identify regions corresponding to features with the most significant leverage scores and calculate their frequency across cohorts. Using the physical coordinates of approximately the top half of frequent regions, we compute the intersection of coordinates for pairs of atlases. Finally, we calculate the overlap coefficient for the top frequent regions in the two atlases using the following formula:


(2) $$\begin{array}{l} \text{Overlap (A,B)}=\frac{\left|A\cap B\right|}{\min\left(\left|A|,|B\right|\right)} \end{array}$$

where A and B are the sets of top frequent regions in the two atlases, this metric modifies the Dice coefficient used by Lawrence et al. (2021). We found our version more pertinent, as region sizes vary significantly between fine-grained atlases, such as the one by Craddock et al., and coarse-grained atlases, such as AAL and HOA. Craddock's fine-grained nature likely contributes to its higher reliability in capturing individual-specific connectivity features compared to coarser parcellations. In fact, finer-grained parcellations can better preserve subtle functional variations that coarser atlases may average out. Liu et al. (2017) showed that high-resolution parcellations improve the differentiation of consciousness states during anesthesia, highlighting their ability to detect finer-scale functional connectivity changes compared to coarser parcellations. Similarly, Wang et al. (2023) developed fine-grained cortical parcellation maps for infants, showing that high-resolution parcellations reveal complex functional developmental patterns, such as changes in local network efficiency and connectivity gradients. These findings support using finer parcellations like the Craddock atlas to identify stable brain signatures across age groups and tasks.

Given these advantages, our results (Table 3) show that the overlap between Craddock and AAL regions is 0.67, 0.71, and 0.71 for rest, movie, and SMT states, respectively, while the overlap between Craddock and HOA regions is 0.56, 0.43, and 0.55. Higher scores indicate greater agreement between regions corresponding to top-leverage scores across atlases.

**Table 3:** This table shows the overlap coefficients between pairs of coordinate sets representing frequent regions associated with top features identified by the leverage score method.

**Atlas-pairs**	**Tasks**		
	**Rest**	**Movie**	**SMT**
Craddock-AAL	0.67	0.71	0.71
Craddock-HOA	0.56	0.43	0.55

Comparisons are made between Craddock-AAL and Craddock-HOA atlas pairs. Results are shown for three tasks: Rest, Movie, and SMT. Higher coefficients indicate greater consistency in identified regions across atlas pairs, demonstrating the reproducibility of individual-specific neuroanatomical signatures.

These results demonstrate that the overlap between AAL and Craddock is consistently higher than between HOA and Craddock, suggesting greater congruence between these two atlases. This difference can be attributed to variations in spatial definitions. While AAL and HOA have nearly the same number of regions, their alignment with Craddock differs. Specifically, the percentage of common voxels between Craddock and AAL (≈90%) is significantly higher than between Craddock and HOA (≈78%), indicating that Craddock's fine-grained functional regions align more closely with AAL's anatomical subdivisions than with HOA's. This greater voxel-level overlap likely explains the observed discrepancy in overlap scores.

To assess the statistical significance of these results, we calculated the average overlap for random features, which was observed to be zero. Our observed overlaps were significantly higher, with a p-value below < 1*e* − 8, substantiating the robustness and reliability of our findings.

These results demonstrate the consistency of signatures across different atlas representations, particularly between Craddock and AAL atlases. This consistency suggests that insights derived from signatures in one atlas also largely apply to other atlases.

## 4 Discussion

Our study provides significant insights into the stability and reproducibility of individual-specific brain signatures across the adult lifespan and different brain atlases.

The key findings of our research lead to several important conclusions: Our leverage score-based feature selection method effectively characterizes individual-specific signals across all age groups, minimizing inter-subject similarity while maximizing intra-subject consistency across tasks. This approach provides a robust tool for identifying unique brain connectivity patterns. We demonstrate the remarkable stability of individual-specific signatures across the adult lifespan (18–87 years), suggesting the existence of core neuroanatomical characteristics that remain stable from early adulthood to old age. This finding is crucial for establishing a baseline of neural features resilient to normal aging. Our consistent results across Craddock, AAL, and HOA parcellations underscore the robustness of these individual-specific patterns, indicating that the identified features represent genuine neurobiological phenomena rather than artifacts of a particular anatomical framework.

The age-resilient features identified in this study have the potential to serve as reliable biomarkers for distinguishing normal aging from pathological neurodegeneration. This could be particularly valuable in the context of the increase in neurodegenerative diseases in an aging population. Our findings suggest that components of individual-specific signatures have age-resilient and age-dependent components. Further, the characterization of changes in the feature sets across age is potentially crucial for understanding age-dependent factors.

While this study provides valuable insights into the stability of individual-specific brain signatures, several limitations must be acknowledged. One consideration is the use of age cohorts of equal size, which ensures statistical comparability but does not fully capture the natural distribution of ages in the population. Although an alternative grouping based on 5-year cohorts yielded similar results, future studies on larger datasets may explore more flexible cohort definitions to further validate our findings. Additionally, our analysis is limited to healthy subjects, restricting the generalizability of our results to clinical populations. Investigating whether similar stability patterns persist in individuals with neurological conditions or other health-related variability would be an important avenue for future research. Finally, while our findings suggest that certain brain regions exhibit stability across age groups, longitudinal studies are necessary to confirm their consistency over time. Establishing whether these regions remain functionally stable across extended periods would provide stronger evidence that they serve as reliable markers of individual-specific brain signatures.

In conclusion, our study validates a robust methodology for characterizing individual brain architecture and provides valuable insights into the stability of these characteristics across adulthood. These findings contribute to a more nuanced understanding of brain organization and aging, with potential applications in research and clinical settings.

## Data Availability

Publicly available datasets were analyzed in this study. This data can be found here: https://cam-can.mrc-cbu.cam.ac.uk/dataset/.
