# Supplementary material for: Characterizing changes to individual-specific brain signature with age
Source: Front Aging Neurosci. 2025 Jul 2;17:1493855. doi: 10.3389/fnagi.2025.1493855 (PMC12263593; doi:10.3389/fnagi.2025.1493855)
Supplement: Supplementary file 1 [file Data_Sheet_1.pdf]

Supplementary Material

1 FEATURE OVERLAPS FOR DIFFERENT ATLASES

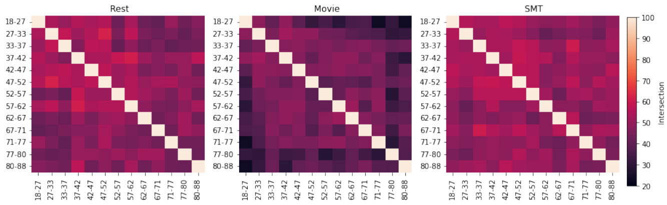

Figure S1. a) AAL.

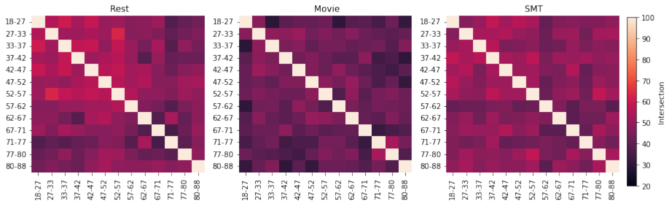

Figure S1. b) HOA.

Figure S1. These heatmaps show the percentage intersection of top features selected using the leverage score method between age groups in the a) AAL and b) HOA atlases, for three tasks: Rest, Movie, and SMT. Each cell represents the percentage of shared top features between two age groups, with warmer colors indicating higher overlap. The subjects are divided into non-overlapping subsets of 50 individuals, arranged by age. The diagonal represents 100% self-intersection. This visualization reveals the consistency of individual-specific neural signatures across different age ranges and cognitive states, demonstrating how brain connectivity patterns evolve or remain stable throughout adulthood under various task conditions.The average percentage intersection across age groups for AAL is 48.9% for Rest, 41% for Movie, and 50.5% for SMT, while for HOA is 48.6% for Rest, 41.3% for MOvie, and 48.1% for SMT.

2 FEATURE OVERLAPS FOR MALE AND FEMALE SUBJECTS

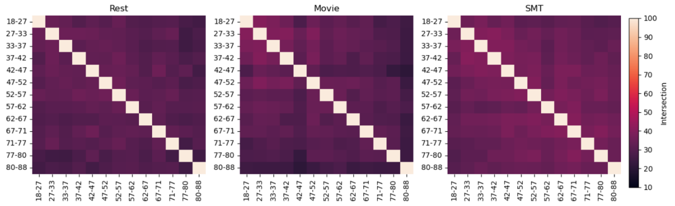

Figure S2. a) Male subjects.

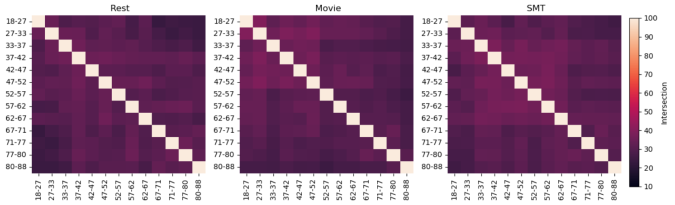

Figure S2. b) Female subjects.

Figure S2. These heatmaps show the percentage intersection of top features selected using the leverage score method between age groups in the a) Male and b) female subjects, based on the Craddock parcellation, for three tasks: Rest, Movie, and SMT. Each cell represents the percentage of shared top features between two age groups, with warmer colors indicating higher overlap. The subjects are divided into non-overlapping subsets of 24 individuals, arranged by age. The diagonal represents 100% self-intersection. This visualization reveals slight differences in feature stability, with males generally showing slightly higher stability in the SMT task than females. However, these differences are not substantial. The average percentage intersection across age groups is 30.8% for males and 30.5% for females in Rest, 30.8% for males and 31% for females in Movie, and 35.3% for males and 32% for females in SMT.

3 STABLE FUNCTIONAL CONNECTIONS ACROSS AGE COHORTS

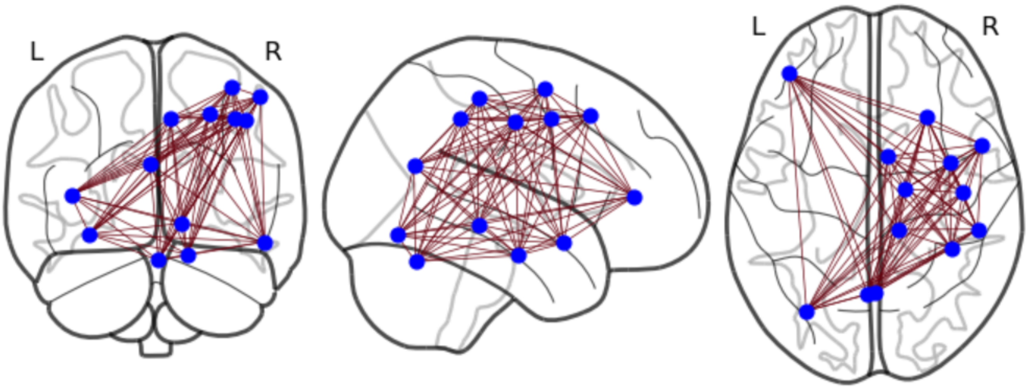

Figure S3. Connectome plot visualizing the stable functional connectivity (FC) features consistently identified across all age cohorts. Nodes represent brain regions, and edges represent stable functional connections. All edges are displayed in blue to indicate consistent presence across lifespan, and node size is proportional to region selection.

4 COHORT-WISE SIMILARITY BETWEEN PAIRS OF SUBJECTS

Task	Age	Feature Set		
		All	LS	Random
Rest	18–27	0.2385	0.0558	0.2307
	27–33	0.2411	0.0378	0.2322
	33–37	0.2438	0.0584	0.2715
	37–42	0.2714	0.0316	0.2349
	42–47	0.2394	0.0424	0.2469
	47–52	0.2460	0.0496	0.2419
	52–57	0.2467	0.0464	0.2207
	56–62	0.2499	0.0590	0.2542
	62–67	0.2379	0.0587	0.2604
	67–71	0.2422	0.0427	0.2585
	71–77	0.2404	0.0565	0.271
	77–80	0.2412	0.0337	0.2295
	80–88	0.2574	0.0308	0.2265
SMT	18–27	0.2299	0.0416	0.2446
	27–33	0.2333	0.0476	0.2294
	33–37	0.2451	0.0386	0.2095
	37–42	0.2315	0.0519	0.2394
	42–47	0.2038	0.0392	0.23230
	47–52	0.2407	0.0397	0.2245
	52–57	0.2392	0.0464	0.2303
	56–62	0.2313	0.0327	0.2351
	62–67	0.2443	0.0481	0.2256
	67–71	0.2414	0.0438	0.2301
	71–77	0.2136	0.0284	0.2308
	77–80	0.2418	0.0381	0.2593
	80–88	0.2162	0.0365	0.2475
Movie	18–27	0.1450	0.0345	0.1866
	27–33	0.1321	0.0367	0.1317
	33–37	0.1326	0.0416	0.1537
	37–42	0.1219	0.0310	0.1479
	42–47	0.1822	0.0323	0.1603
	47–52	0.1402	0.0313	0.1637
	52–57	0.1210	0.0382	0.1622
	56–62	0.1417	0.0366	0.1396
	62–67	0.1585	0.0338	0.1376
	67–71	0.1208	0.0297	0.1561
	71–77	0.1434	0.0252	0.1343
	77–80	0.1667	0.0305	0.1545
	80–88	0.1454	0.0317	0.1239

Table S1. This table shows the average similarity between pairs of subjects when they are performing one of the three tasks (Rest, SMT, and Movie). In each case, we present results for a) all features, b) top 1000 leverage-score (LS) features, and c) randomly selected features, for each cohort of subjects. The consistent low similarity for top LS shows that our feature selection approach ensures high dissimilarity across subjects. Here, we use Pearson Correlation as a measure of similarity

Task-Pairs	Age	Feature Set		
		All	LS	Random
Rest-SMT	18–27	0.4470	0.4429	0.4191
	27–33	0.4767	0.4356	0.4462
	33–37	0.4809	0.5142	0.4745
	37–42	0.4753	0.4586	0.4572
	42–47	0.4822	0.4163	0.4592
	47–52	0.4701	0.5076	0.4299
	52–57	0.4110	0.0464	0.4397
	56–62	0.4607	0.4475	0.4598
	62–67	0.4815	0.3953	0.4893
	67–71	0.4227	0.4753	0.4571
	71–77	0.4631	0.4685	0.4617
	77–80	0.4659	0.4520	0.4362
	80–88	0.4991	0.4946	0.4760
Rest-Movie	18–27	0.4407	0.4105	0.4056
	27–33	0.4640	0.4099	0.4440
	33–37	0.4426	0.4134	0.4239
	37–42	0.4679	0.4025	0.4119
	42–47	0.4603	0.4046	0.4716
	47–52	0.4033	0.4141	0.4326
	52–57	0.4015	0.0464	0.4147
	56–62	0.4467	0.4123	0.4304
	62–67	0.4333	0.4099	0.4610
	67–71	0.4399	0.4316	0.4459
	71–77	0.4385	0.4015	0.4212
	77–80	0.3993	0.3908	0.4544
	80–88	0.4332	0.3854	0.4388

Table S2. This table shows the average similarity between pairs of FCs drawn from the same subject performing different tasks. In each case, we present results for a) all features, b) top 1000 leverage-score (LS) features, and c) randomly selected features, for each cohort of subjects. When compared to Table S3, the higher PCs for LS features demonstrate that within-subject similarity is strongly encoded in our top features.

5 RESULTS AFTER REMOVING FEATURES WITH INSUFFICIENT COVERAGE

List of Regions: The following are the Craddock-840 region IDs with insufficient coverage: 41, 64, 67, 71, 74, 91, 95, 101, 110, 122, 168, 174, 178, 182, 207, 304, 305, 344, 347, 367, 375, 414, 477, 497, 501, 546, 576, 602, 638, 649, 660, 737, 741, 821, 830.

Task	Feature Set		
	All	LS	Random
Rest	0.2559 ± 0.0132	0.0441 ± 0.011	0.2568 ± 0.0188
SMT	0.2342 ± 0.0190	0.0435 ± 0.0086	0.2229 ± 0.0231
Movie	0.1501 ± 0.0254	0.0348 ± 0.0069	0.1512 ± 0.0287

Table S3. This table shows the average similarity between pairs of subjects when they are performing one of the three tasks (Rest, SMT, and Movie) after excluding regions with insufficient coverage. In each case, we present results for a) all features, b) top 1000 leverage-score (LS) features, and c) randomly selected features. The consistent low similarity for top LS shows that our feature selection approach ensures high dissimilarity across subjects. Here, we use Pearson Correlation as a measure of similarity

Task-Pairs	Feature Set		
	All	LS	Random
Rest-SMT	0.4511 ± 0.0200	0.4558 ± 0.0302	0.4513 ± 0.0226
Rest-Movie	0.4428 ± 0.0218	0.4253 ± 0.0206	0.4432 ± 0.0217

Table S4. This table shows the average similarity between pairs of FCs drawn from the same subject performing different tasks after removing regions with insufficient coverage. In each case, we present results for a) all features, b) top 1000 leverage-score (LS) features, and c) randomly selected features. When compared to Table S3, the higher PCs for LS features demonstrate that within-subject similarity is strongly encoded in our top features.
